# Dyslipidemia in children with chronic kidney disease—findings from the Cardiovascular Comorbidity in Children with Chronic Kidney Disease (4C) study

**DOI:** 10.1007/s00467-024-06389-3

**Published:** 2024-05-08

**Authors:** Francesca Mencarelli, Karolis Azukaitis, Marietta Kirchner, Aysun Bayazit, Ali Duzova, Nur Canpolat, Ipek Kaplan Bulut, Lukasz Obrycki, Bruno Ranchin, Rukshana Shroff, Salim Caliskan, Cengiz Candan, Alev Yilmaz, Zeynep Birsin Özcakar, Harika Halpay, Aysel Kiyak, Hakan Erdogan, Jutta Gellermann, Ayse Balat, Anette Melk, Franz Schaefer, Uwe Querfeld

**Affiliations:** 1 Pediatric Nephrology Unit, Department of Pediatrics, S. Orsola-Malpighi Hospital, University of Bologna, Bologna, Italy; 2 Clinic of Pediatrics, Institute of Clinical Medicine, Faculty of Medicine, Vilnius University, Vilnius, Lithuania; 3 Institute for Medical Biometry and Informatics, University of Heidelberg, Heidelberg, Germany; 4 Department of Pediatric Nephrology, Cukurova University, Adana, Turkey; 5 Division of Pediatric Nephrology, Faculty of Medicine, Hacettepe University, Ankara, Turkey; 6 Department of Pediatric Nephrology, Faculty of Medicine, Istanbul University-Cerrahpasa, Istanbul, Turkey; 7 Division of Pediatric Nephrology, Department of Pediatrics, Faculty of Medicine, Ege University, Izmir, Turkey; 8 Department of Nephrology and Arterial Hypertension, Children’s Memorial Health Institute, Warsaw, Poland; 9 Pediatric Nephrology Unit, Hôpital Femme Mère Enfant, Hospices Civils de Lyon, Université de Lyon, Lyon, France; 10 UCL Great Ormond Street Institute of Child Health, London, UK; 11 Division of Pediatric Nephrology, Göztepe Hospital, Istanbul Medeniyet University, Istanbul, Turkey; 12 Faculty of Medicine, Istanbul University, Istanbul, Turkey; 13 Division of Pediatric Nephrology, Department of Pediatrics, School of Medicine, Ankara University, Ankara, Turkey; 14 Department of Pediatric Nephrology, Faculty of Medicine, Marmara University, Istanbul, Turkey; 15 Division of Pediatric Nephrology, Department of Pediatrics, Bakirkoy Children’s Hospital, Istanbul, Turkey; 16 Division of Pediatric Nephrology, Faculty of Medicine, Uludağ University, Bursa, Turkey; 17 Department of Pediatric Gastroenterology, Nephrology and Metabolic Diseases, Charité University Hospital, Berlin, Germany; 18 Department of Pediatric Nephrology, Gaziantep University, Gaziantep, Turkey; 19 Department of Kidney, Liver and Metabolic Diseases, Hannover Medical School, Hannover, Germany; 20 Pediatric Nephrology Division, Center for Pediatrics and Adolescent Medicine, Heidelberg, Germany

**Keywords:** Children, Chronic kidney disease, Dyslipidemia, Fasting, Albuminuria

## Abstract

**Background:**

Dyslipidemia is an important and modifiable risk factor for CVD in children with CKD.

**Methods:**

In a cross-sectional study of baseline serum lipid levels in a large prospective cohort study of children with stage 3–5 (predialysis) CKD, frequencies of abnormal lipid levels and types of dyslipidemia were analyzed in the entire cohort and in subpopulations defined by fasting status or by the presence of nephrotic range proteinuria. Associated clinical and laboratory characteristics were determined by multivariable linear regression analysis.

**Results:**

A total of 681 patients aged 12.2 ± 3.3 years with a mean eGFR of 26.9 ± 11.6 ml/min/1.73 m^2^ were included. Kidney diagnosis was classified as CAKUT in 69%, glomerulopathy in 8.4%, and other disorders in 22.6% of patients. Nephrotic range proteinuria (defined by a urinary albumin/creatinine ratio > 1.1 g/g) was present in 26.9%. Dyslipidemia was found in 71.8%, and high triglyceride (TG) levels were the most common abnormality (54.7%). Fasting status (38.9%) had no effect on dyslipidemia status. Except for a significant increase in TG in more advanced CKD, lipid levels and frequencies of dyslipidemia were not significantly different between CKD stages. Hypertriglyceridemia was associated with younger age, lower eGFR, shorter duration of CKD, higher body mass index (BMI-SDS), lower serum albumin, and higher diastolic blood pressure.

**Conclusions:**

Dyslipidemia involving all lipid fractions, but mainly TG, is present in the majority of patients with CKD irrespective of CKD stage or fasting status and is significantly associated with other cardiovascular risk factors.

**Graphical abstract:**

**Figure:**
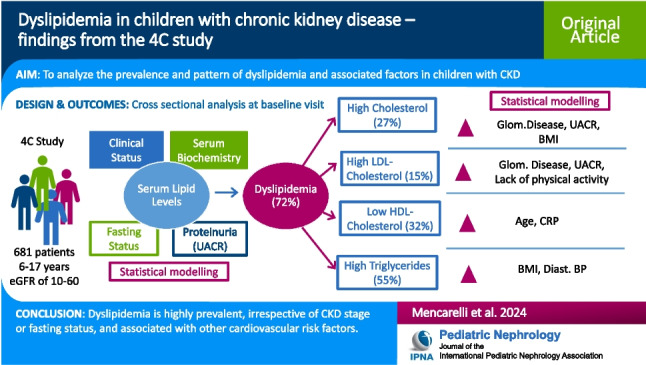
A higher resolution version of the Graphical abstract is available as Supplementary information

**Supplementary Information:**

The online version contains supplementary material available at 10.1007/s00467-024-06389-3.

## Introduction

Dyslipidemia is a common metabolic complication of chronic kidney disease (CKD). Prior to dialysis or transplantation, CKD is typically associated with increased levels of triglycerides (TGs) and a decrease in high-density lipoprotein cholesterol (HDL-C), while levels of total cholesterol (CHOL) and low-density lipoprotein cholesterol (LDL-C) are variable [1–3]. A variety of factors, such as primary kidney disease, CKD stage, degree of proteinuria, medications, diet, the presence of malnutrition or obesity, and gender effects, have further modifying effects on the lipid profile [4]. Marked hyperlipidemia with highly elevated levels of CHOL and LDL-C may be found in patients with CKD and nephrotic syndrome or nephrotic range proteinuria [2, 5].

Dyslipidemia is a traditional risk factor for cardiovascular disease (CVD), the leading cause of morbidity and mortality in patients with CKD, including children [6]. Cross-sectional data from large cohort studies in children with CKD show a high prevalence of dyslipidemia (35–65%), significantly associated with the degree of proteinuria, obesity, and diminishing kidney function [5, 7]. Thus, dyslipidemia is an important and modifiable risk factor for CVD in children with CKD, a pediatric population with an extremely high cardiovascular risk [8].

Pediatric cohort studies provide the unique opportunity to study the impact of CKD in a population without diabetes or age- or lifestyle-related comorbidities. Here, we performed a cross-sectional investigation to analyze the prevalence and pattern of dyslipidemia, as well as associated patient-related and CKD-related factors, in a large cohort of children enrolled in the prospective Cardiovascular Comorbidity in Children with Chronic Kidney Disease (4C) study.

## Methods

### Study setting and design

The prospective 4C study (ClinicalTrials.gov NCT01046448) observed 704 children aged 6–17 years with predialysis CKD stage 3–5 and a baseline eGFR of 10–60 ml/min/1.73 m^2^, who were enrolled between 2010 and 2012 at 55 pediatric nephrology units in 12 European countries and followed until the end of 2018. The study was approved by the Ethics Committee of the University of Heidelberg (S-032/2009) and the Institutional Review Boards of each participating center. Written informed consent was obtained from all parents and participants, where appropriate. Patients underwent standardized biannual clinical evaluations including the collection of blood and urine samples and an annual cardiovascular examination; a detailed study protocol and an analysis of the cardiovascular phenotype of this patient cohort have been published [9, 10].

Patients with at least one serum lipid value available at baseline were included in the present study. Patients taking lipid-lowering drugs were excluded. Fasting status was recorded at each visit. The baseline data of the different lipid fractions (TG, CHOL, LDL-C, HDL-C) was analyzed for associations with clinical and laboratory variables. Subgroup analyses were performed in children with or without nephrotic range proteinuria and in fasting patients.

### Definitions

Dyslipidemia was defined as the presence of at least one abnormal serum lipid level (CHOL, LDL-C, HDL-C, TG) according to published age-dependent cut-off values (Supplemental Table 1) [11].

The various forms of dyslipidemia were categorized according to their baseline serum levels as (a) no dyslipidemia, (b) isolated dyslipidemia (one abnormal), and (c) multiple dyslipidemia (multiple abnormal measurements, i.e., at least 2 lipids abnormal).

Primary kidney diseases were classified as CAKUT (congenital abnormalities of the kidney and urinary tract), glomerular diseases, and others.

Body mass index (BMI), height, and office blood pressure (median of three oscillometric measurements) were normalized by the calculation of standard deviation scores (SDSs) based on reference values for healthy children [12, 13]. To analyze the impact of nutritional status, baseline lipid levels were stratified by BMI categories (underweight, BMI-SDS <  − 2; normal, − 2 ≤ BMI-SDS < 1; overweight, 1 ≤ BMI-SDS < 2; obese, BMI-SDS ≥ 2) [15].

The albumin/creatinine ratio in spot urine was used to quantify proteinuria as recommended by KDIGO guidelines [14]. There is currently no standard definition for nephrotic range albuminuria in children [15]. A urinary protein excretion of > 1 g/m^2^/d and > 0.5 g/d is the accepted cut-off for nephrotic range proteinuria in children and adults, respectively. It has been shown that a urine total protein excretion of 3.5 g/d translates to 2.2 g/g for the urine albumin/creatinine ratio (UACR) in adults [16, 17]. Since 3.5 g/d for the average adult body surface area of 1.73 m^2^ amounts to 2 g/m^2^/d, we adjusted the adult definition (UACR of 2.2 g/g) to the pediatric definition of 1 g/m^2^/d, arriving at a UACR of 1.1 g/g, which was used as the cut-off to define nephrotic range albuminuria.

### Laboratory methods

Estimated GFR (eGFR) was calculated using the updated Schwartz equation [18]. All blood and urine samples were collected at a regular visit in the outpatient clinic and measured by fully automated methods at a central laboratory (Synlab, Heidelberg) using stored serum samples collected at each 6-monthly visit. Urinary albumin and creatinine were measured in spot urine samples. All lipids were measured directly by enzymatic test on a Siemens Advia 2400 analyzer, including LDL-C (and not calculated with the widely used Friedewald formula, which should not be used in non-fasting patients).

### Statistical analysis

Clinical and laboratory characteristics for the study population are described using mean (SD), median (IQR), or frequencies for continuous and categorical variables, respectively. Differences between non-nephrotic vs. nephrotic range proteinuria groups were analyzed using a *t*-test, Mann–Whitney *U* test, or chi-squared test, respectively. Abnormal lipid levels at baseline and multiple dyslipidemia based on all four lipids are described by frequencies in the total sample and stratified by CKD stage where differences between stages were analyzed by the chi-squared test. Additionally, differences in means between CKD stage categories or between BMI categories were analyzed by analysis of variance (ANOVA) for each lipid. Frequencies of TG vs. HDL dyslipidemia are shown in four-fold tables with *p*-values based on chi-squared tests to compare the distribution of TG and HDL dyslipidemia. To explore linear relationships, Pearson’s correlation coefficients were calculated among baseline lipids or among two clinical variables of interest (e.g., albumin and UACR) where skewed distributed variables were log-transformed.

Multivariable linear regression analysis was performed to identify clinical and laboratory characteristics associated with baseline lipid levels. In the full model, all candidate variables included were considered to be biologically relevant. Missing values were not imputed, and an available case analysis was performed. To reduce the model, variable selection was applied with backward selection based on the Akaike information criterion (AIC). Regression coefficients with 95% confidence intervals (CI) and *p*-values are shown.

Statistical analysis was performed using SAS Version 9.4. *p*-values < 0.05 were considered statistically significant and were interpreted descriptively due to the exploratory nature of all analyses.

## Results

### Baseline characteristics

**Fig. 1:**
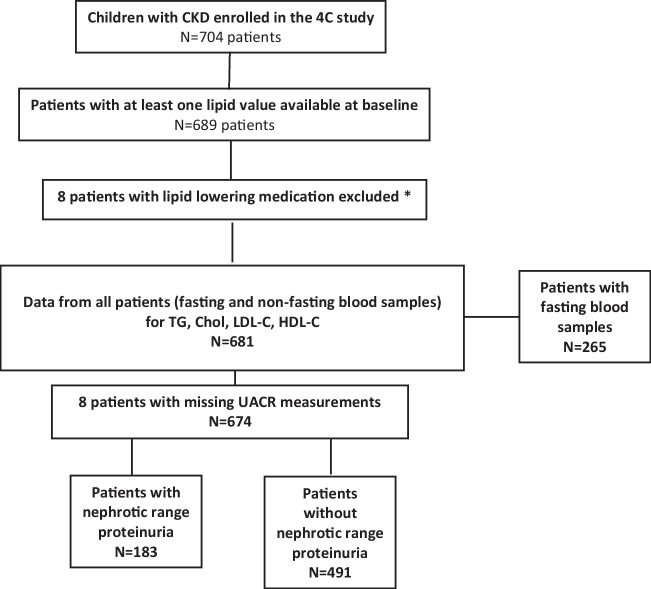
Patient and observation selection flow chart. The albumin/creatinine ratio (UACR) in spot urine was used to quantify proteinuria. A UACR of 1.1 g/g was defined as the cut-off for nephrotic range proteinuria in children. *Lipid-lowering medication: statins (*n* = 7; in 2 cases in combination with ezetimibe), fibrate (*n* = 1)

A total of 689 patients had at least one lipid measurement at enrollment; eight patients taking lipid-lowering medication were excluded. Thus, 681 patients (611 Caucasian = 89.7%; 448 boys = 65.8%) aged 12.2 ± 3.3 years meeting the eligibility criteria, with a median eGFR of 26.9 ± 11.6 ml/min/1.73 m^2^, were included in the analysis (Fig. 1). Baseline characteristics of all patients are summarized in Table 1. Primary kidney diagnosis was CAKUT in 69% (*n* = 470), glomerulopathy in 8.4% (*n* = 57), and others in 22.6% (*n* = 154) patients. The mean BMI was 18.4 ± 3.9 kg/m^2^, 18.4% (*n* = 125) were overweight, and 5.3% (*n* = 36) patients were obese. Of all patients, 265 (38.9%) were fasting at the time of examination, and 416 (61.1%) were not fasting.

**Table 1:** Clinical characteristics of the study population and subgroups with or without nephrotic range proteinuria

	All (*n* = 681)	Nephrotic range proteinuria (*n* = 183)	Non-nephrotic range proteinuria (*n* = 491)	*P*
Age (yrs)	12.2 (3.3)	12.8 (3.2)	11.9 (3.4)	0.003
Sex (% boys)	448 (65.8%)	116 (63.4%)	329 (67.0%)	0.378
Caucasian ethnicity	611 (89.7%)	163 (89.1%)	441 (89.8%)	0.778
Primary kidney diagnosis				< 0.001
CAKUT	470 (69.0%)	113 (63.7%)	354 (72.1%)	
Glomerular	57 (8.4%)	33 (18.0%)	24 (4.9%)	
Other	154 (22.6%)	37 (20.2%)	113 (23.0%)	
Pubertal status-pubertal	303 (44.9%)	94 (51.6%)	204 (42.0%)	0.025
Height (cm)	140.9 (20.2)	142.9 (19.5)	140.1 (20.4)	0.110
SDS	− 1.35 (1.36)	− 1.51 (1.25)	− 1.29 (1.39)	0.061
BMI (kg/m^2^)	18.4 (3.9)	18.6 (3.6)	18.4 (4.0)	0.552
SDS	0.10 (1.28)	0.15 (1.24)	0.09 (1.29)	0.621
Obese (%)	36 (5.3%)	10 (5.5%)	26 (5.3%)	0.940
Overweight (%)	125 (18.4%)	33 (18.0%)	91 (18.5%)	0.881
Waist-to-hip ratio	0.92 (0.12)	0.90 (0.11)	0.93 (0.12)	0.005
Waist-to-height ratio	0.48 (0.07)	0.48 (0.07)	0.49 (0.07)	0.259
eGFR (ml/min/1.73 m^2^)	26.9 (11.6)	22.1 (9.5)	28.7 (11.8)	< 0.001
Time since CKD stage II (years)	6.1 (4.6)	5.3 (4.1)	6.4 (4.7)	0.010
Bicarbonate (mmol/l)	21.3 (3.6)	20.7 (4.2)	21.5 (3.4)	0.010
CRP (mg/dl)	0.55 (1.99)	0.60 (2.6)	0.54 (2.0)	0.400
Hemoglobin (g/dl)	11.7 (1.6)	11.5 (1.7)	11.8 (1.6)	0.025
Serum albumin (g/l)	40.0 (5.5)	35.0 (6.9)	40.5 (3.9 g)	< 0.001
UACR (g/g)	0.33 (1.14)	2.29 (1.93)	0.17 (0.38)	< 0.001
Systolic BP SDS	0.80 (1.35)	1.07 (1.50)	0.71 (1.29)	0.002
Diastolic BP SDS	0.69 (1.08)	1.0 (1.17)	0.58 (1.03)	< 0.001
Physical activity				< 0.001
< 1 h per week	159 (23.8%)	61 (33.7%)	95 (19.8%)	
1–4 h per week	228 (34.2%)	52 (28.7%)	172 (35.9%)	
> 4 h per week	280 (42.0%)	68 (37.6%)	212 (44.3%)	

Nephrotic range proteinuria was defined as urinary albumin/creatinine ratio > 1.1 g/g (UACR; measurements available in *N* = 674). CRP and UACR expressed as median and IQR; all other measurements as mean and SD. UACR (g/g) = 10*ualb (mg/dl)/ucrea (mg/l). There were 7 patients with missing UACR measurements (not permitting classification for proteinuria). Obesity: BMI-SDS ≥ 2. Overweight: 1 ≤ BMI-SDS < 2. *P* denotes significance of differences between subgroups with or without nephrotic range proteinuria.

Data were further analyzed in the entire cohort and in the subgroups of patients with (*n* = 183; 26.8%) or without (*n* = 491; 72.2%) nephrotic range proteinuria as defined by a UACR of > 1.1 g/g. Serum lipid levels at baseline in all patients and in the subgroups are shown in Table 2. The pattern of “uremic dyslipidemia” characterized by high TG, low-normal HDL-C, and normal CHOL and LDL-C levels was seen in patients with or without nephrotic range proteinuria. Lipid levels showed several significant intercorrelations (Supplemental Table 2). Furthermore, lipids, with the exception of TG, were not significantly different between fasting and non-fasting patients (Supplemental Table 3). The TG and HDL-C levels were significantly different between BMI categories, with a steady increase in TG and a decrease in HDL-C with increasing BMI, whereas CHOL and LDL-C levels were similar (Supplemental Table 4).

**Table 2:** Baseline lipid levels (all in mg/dl) of the study population and subgroups

A. All patients				
	All (*n* = 681)	Nephrotic range proteinuria (*n* = 183)	Non-nephrotic range proteinuria (*n* = 491)	*p*-value
Total cholesterol				
Mean (SD)	180 (48)	199 (62)	172 (37)	< 0.001
Median (IQR)	173 (55)	185 (72)	171 (50)	
HDL cholesterol				
Mean (SD)	48 (14)	49 (16)	48(14)	0.676
Median (IQR)	46 (19)	46 (20)	46 (18)	
LDL cholesterol				
Mean (SD)	98 (39)	113 (51)	92 (30)	< 0.001
Median (IQR)	93 (46)	100 (53)	91 (42)	
Triglycerides				
Mean (SD)	147 (86)	170 (101)	138 (78)	< 0.001
Median (IQR)	126 (84)	142 (107)	119 (78)	
B. Fasting patients				
	All (*n* = 265)	Nephrotic-range proteinuria (*n* = 85)	Non-nephrotic range proteinuria (*n* = 177)	*p*-value
Total cholesterol				
Mean (SD)	180 (54)	198 (68)	169 (38)	< 0.001
Median (IQR)	172 (58)	182 (67)	169 (52)	
HDL cholesterol				
Mean (SD)	48 (15)	48 (16)	47 (14)	0.749
Median (IQR)	46 (19)	45 (22)	46 (18)	
LDL cholesterol				
Mean (SD)	100 (44)	115 (55)	91 (31)	< 0.001
Median (IQR)	94 (47)	102 (50)	90 (40)	
Triglycerides				
Mean (SD)	137 (75)	154 (83)	129 (70)	0.015
Median (IQR)	123 (80)	135 (96)	115 (74)	

*n* = 674 (panel A) and *n* = 262 (panel B) patients in the subgroups (nephrotic vs. non-nephrotic) due to 7 (panel A) (3 for panel B) with missing data for albuminuria. *P*-values are based on a two-sample *t*-test.

### Abnormal lipid levels, types, and frequency of dyslipidemia

Hypertriglyceridemia was the most frequent abnormality, found in 54.7% of the study population. Abnormal serum concentrations of HDL-C (31.9%), CHOL (27.1%), and LDL-C (15.2%) were also frequent (Table 3).

**Table 3:** Lipids and frequency of abnormal levels at baseline stratified by CKD stage

	All	CKD stage 3	CKD stage 4	CKD stage 5	*p-value
Total cholesterol (*n***)**	679	249	322	108	
Mean (± SD; mg/dl)	180 (48)	177 (45)	183 (49)	178 (52)	0.294
Normal (n, %)	495 (72.9%)	181 (72.7%)	233 (72.4%)	81 (75.03%)	0.863
Abnormal (n, %)	184 (27.1%)	68 (27.3%)	89 (27.6%)	27 (25.0%)	
HDL cholesterol	681	249	324	108	
Mean (± SD; mg/dl)	48 (14)	49 (14)	47 (15)	46 (16)	0.088
Normal	464 (68.1%)	188 (75.5%)	207 (63.9%)	69 (63.9%)	0.007
Abnormal	217 (31.9%)	61 (24.5%)	117 (36.1%)	39 (36.1%)	
LDL cholesterol	678	249	322	107	
Mean (± SD; mg/dl)	98 (39)	96 (37)	101 (40)	96 (41)	0.217
Normal	575 (84.8%)	211 (84.7%)	268 (83.2%)	96 (89.7%)	0.269
Abnormal	103 (15.2%)	38 (15.3%)	54 (16.8%)	11 (10.3%)	
Triglycerides	680	249	324	107	
Mean (± SD; mg/dl)	147 (86)	134 (78)	154 (89)	154 (92)	0.015
Normal	308 (45.3%)	130 (52.2%)	135 (41.7%)	43 (40.2%)	0.022
Abnormal	372 (54.7%)	119 (47.8%)	189 (58.3%)	64 (59.8%)	

CKD stage was modeled as a categorical variable

*p-value is based on the chi-squared test for dichotomized lipid variables (normal vs. abnormal) and on ANOVA for continuous lipid variables

In the entire cohort and in the subgroup of fasting patients, those with nephrotic range proteinuria had significantly elevated levels of CHOL, TG, and LDL-C, while HDL-C levels were similar. Serum albumin levels were significantly lower in patients with nephrotic range proteinuria, but largely within the normal range and only weakly correlated with log-transformed UACR (*r* =  − 0.40; *p* < 0.0001).

Except for a significant increase in TG in more advanced CKD, lipid levels and frequencies of dyslipidemia were not significantly different between CKD stages in the entire cohort (Table 3). In fasting patients, all lipid levels including TG were not significantly different between CKD stages (Supplemental Table 5).

Increased TG levels were frequently accompanied by low levels of HDL-C, especially in patients with nephrotic range proteinuria (Table 4).

**Table 4:** Prevalence of normal and abnormal TG and HDL-C levels

	All	HDL-C low	HDL-C normal	*p*-value
A. Total study population				
High vs. normal TG (*n*; %)				
Normal	308 ( 45.3%)	64 ( 29.5%)	244 ( 52.7%)	< 0.001
Abnormal	372 ( 54.7%)	153 ( 70.5%)	219 ( 47.3%)	
Missing	1	0	1	
B. Patients with nephrotic range proteinuria				
High vs. normal TG (*n*; %)				
Normal	65 ( 35.5%)	14 ( 23.0%)	51 ( 41.8%)	0.012
Abnormal	118 ( 64.5%)	47 ( 77.0%)	71 ( 58.2%)	
C. Patients without nephrotic range proteinuria				
High vs. normal TG (*n*; %)				
Normal	240 ( 49.0%)	50 ( 32.5%)	190 ( 56.5%)	< 0.001
Abnormal	250 ( 51.0%)	104 ( 67.5%)	146 ( 43.5%)	
Missing	1	0	1	

The frequencies of different types of dyslipidemia at the various CKD stages were not significantly different (Table 5) in the entire cohort as well as in fasting patients (Supplemental Table 6). Irrespective of the CKD stage, the predominant lipid abnormality was high TG levels, followed by low HDL-C, high CHOL, and high LDL-C.

**Table 5:** Types of dyslipidemia and frequency at baseline stratified by CKD stage

	All (n; %)	CKD stage 3	CKD stage 4	CKD stage 5	*p*-value
Dyslipidemia					
No dyslipidemia	190 (27.6%)	81 (32.5%)	77 (23.8%)	32 (29.4%)	0.102
Dyslipidemia	489 (71.8%)	168 (67.5%)	246 (75.9%)	75 (64.4%)	
Isolated vs. multiple dyslipidemia					
No dyslipidemia	190 (27.9%)	81 (32.5%)	77 (23.8%)	32 (29.6%)	0.312
Isolated dyslipidemia	220 (32.3%)	79 (31.7%)	110 (34.0%)	31 (28.7%)	
Abnormal lipid					
Isolated high Chol	28 (12.7%)	14 (17.7%)	12 (10.9%)	2 (6.5%)	0.242
Isolated low HDL-C	59 (26.8%)	19 (24.1%)	34 (30.9%)	6 (19.4%)	
Isolated high TG	133 (60.5%)	46 (58.2%)	64 (58.2%)	23 (74.2%)	
Isolated high LDL-C	0				
Multiple dyslipidemia	268 (39.4%)	89 (35.7%)	135 (41.7%)	44 (40.7%)	
Number of abnormal lipids: multiple dyslipidemia					
2	170 (63.4%)	63 (70.8%)	80 (59.3%)	27 (61.4%)	0.264
3	77 (28.7%)	23 (25.8%)	42 (31.1%)	12 (27.3%)	
4	21 (7.8%)	3 (3.4%)	13 (9.6%)	5 (11.4%)	

Dyslipidemia could not be classified due to missing values in 3 patients. CKD stage was modeled as a categorical variable. *p*-values are based on the chi-squared test

### Factors associated with lipid levels and dyslipidemia at baseline

To further analyze factors associated with lipid levels at baseline, multiple linear regression models were constructed for each lipid with covariates, including the fasting status (Table 6). Fasting status had a significant effect only for TG. Serum albumin was the only variable significantly associated with all lipid levels. Glomerular disease and the BMI-SDS had a strong impact on CHOL, LDL-C, and TG levels. To investigate whether the same factors were associated with dyslipidemia, multivariable logistic regression models were constructed for the status of dyslipidemia for each lipid (Table 7). Fasting status had no significant effect on dyslipidemia status. The risk for low HDL-C levels was much lower for girls than for boys. Proteinuria was associated with increased levels of CHOL, LDL-C, and HDL-C, whereas hsCRP had an inverse association with CHOL and HDL-C. The BMI-SDS was associated with abnormal CHOL and TG levels and the presence of glomerular disease with increased levels of CHOL and LDL-C. Hypertriglyceridemia was associated with younger age, lower eGFR, shorter duration of CKD, higher body mass index (BMI-SDS), lower serum albumin, and higher diastolic blood pressure.

**Table 6:** Baseline correlates of lipid levels

	Cholesterol (*n* = 630)		LDL cholesterol (*n* = 629)		HDL cholesterol (*n* = 632)		Triglycerides (*n* = 631)	
	Beta [95%CI]	*p*-value	Beta [95%CI]	*p*-value	Beta [95%CI]	*p*-value	Beta[95%CI]	*p*-value
Age (yrs)	− 1.77 [− 2.79, − 0.76]	0.0007	− 1.21 [− 2.06, − 0.38]	0.0046	− 0.77 [− 1.03, − 0.44]	< 0.0001	n.i	
Sex (ref male)	n.i		n.i		3.82 [1.50, 6.11]	0.0013	− 14.02 [− 27.60, − 0.45	0.0429
Glomerular disease (ref CAKUT)	38.19 [25.34, 51.05]	< 0.0001	28.27 [17.67, 38.85]	< 0.0001	n.i		37.92 [14.07, 61.79]	0.0019
Other diagnosis (ref CAKUT)	12.13 [3.98, 20.28]	0.0036	8.83 [2.12, 15.54]	0.0100	n.i		2.18 [− 13.49, 17.86]	0.7846
eGFR (ml/min/1.73 m^2^)	n.i		n.i		0.15 [0.05, 0.25]	0.0040	− 0.74 [− 1.28, − 0.20]	0.0075
Time since CKD2 (years)	n.i		n.i		0.54 [0.29, 0.79]	< 0.0001	− 1.30 [− 2.78, 0.18]	0.0861
Log UACR	6.20 [4.11, 8.29]	< 0.0001	4.39 [2.67, 2.11]	< 0.0001	1.41 [0.70, 2.12]	0.0001	n.i	
Log hsCRP	− 2.74 [− 4.87, − 0.60]	0.0120	− 1.38 [− 3.14, 0.38]	0.1232	− 1.34 [− 2.02, − 0.66]	0.0001	− 5.52 [− 9.52, − 1.53]	0.0068
BMI-SDS	3.89 [1.26, 6.52]	0.0038	2.21 [− 0.05, 4.39]	0.0449	n.i		10.59 [5.61, 15.57]	< 0.0001
Serum albumin	− 1.22 [− 1.93, − 0.50]	0.0009	− 1.05 [− 1.64, − 0.47]	0.0005	0.49 [0.27, 0.71]	< 0.0001	− 3.68 [− 4.92, − 2.45]	< 0.0001
Diastolic BP SDS	n.i		n.i		n.i		8.31 [2.50, 14.12]	0.0052
Lack of physical activity (ref at least 1 h per week)	9.89 [1.88, 17.89]	0.0156	7.46 [0.87, 14.06]	0.0267	n.i		n.i	
Serum bicarbonate	1.66 [0.71, 2.60]	0.0006	1.35 [0.56, 2.14]	0.0008	0.26 [− 0.05, 0.56]	0.0966	n.i	
Serum hemoglobin	n.i		n.i		n.i		n.i	
Non-fasting status (ref fasting)	− 0.52 [− 7.47, 6.43]	0.8831	− 3.02 [− 8.75, 2.72]	0.3020	− 0.90 [− 3.12, 1.31]	0.4238	17.78 [4.87, 30.68]	0.0070

Multivariable linear regression for lipids (continuous). Biologically relevant variables were considered candidate variables; all candidate variables are shown in the table with “n.i.” indicating “not included.” Since the variables age and puberty as well as waist-to-height ratio and BMI-SDS are correlated, only one of these variables (age, BMI-SDS) was included in the model. Final models after variable selection; backward selection based on AIC. Fasting status forced in the model

*n.i.* not included in the final model, *CI* confidence interval, *Beta* regression coefficient, *ref* reference category, *UACR* urinary albumin/creatinine ratio

**Table 7:** Baseline correlates of dyslipidemia

	High cholesterol (*n* = 630)		High LDL cholesterol (*n* = 629)		Low HDL cholesterol (*n* = 632)		High triglycerides (*n* = 631)	
	OR [95%CI]	*p*-value	OR [95%CI]	*p*-value	OR [95%CI]	*p*-value	OR [95%CI]	*p*-value
Age (yrs)	0.93 [0.88, 0.99]	0.0137	0.91 [0.84, 0.98]	0.0100	1.09 [1.03, 1.15]	0.0025	0.87 [0.82, 0.91]	< 0.0001
Sex (ref male)	n.i		n.i		0.43 [0.29, 0.65]	< 0.0001	n.i	
Glomerular disease (ref CAKUT)	5.02 [2.65, 9.49]	< 0.0001	5.07 [2.47, 10.42]	< 0.0001	n.i		1.80 [0.90, 3.59]	0.0956
Other diagnosis (ref CAKUT)	1.43 [0.90, 2.26.]	0.1262	1.36 [0.76, 2.45]	0.3056	n.i		0.75 [0.49, 1.14]	0.1790
eGFR (ml/min/1.73 m^2^)	1.02 [1.00, 1.04]	0.0595	n.i		0.97 [0.95, 0.99]	0.0007	0.98 [0.97, 0.99]	0.0293
Time since CKD2 (years)	n.i		n.i		0.92 [0.88, 0.96]	0.0002	0.94 [0.91, 0.99]	0.0076
Log UACR	1.38 [1.22 1.57]	< 0.0001	1.22 [1.04, 1.43]	0.0159	0.84 [0.74, 0.94]	0.0032	n.i	
Log hsCRP	0.85[0.74, 0.96]	0.0109	n.i		1.24[1.11, 1.39]	0.0002	n.i	
BMI-SDS	1.26 [1.07, 1.48]	0.0057	1.21 [0.99, 1.49]	0.0577	n.i		1.20 [1.05, 1.37]	0.0090
Serum albumin	n.i		0.95 [0.91, 0.99]	0.0274	0.93 [0.89, 0.96]	0.0001	0.95 [0.92, 0.99]	0.0078
Diastolic BP -SDS	n.i		n.i		n.i		1.25 [1.06, 1.48]	0.0069
Lack of physical activity (ref ≥ 1 h per week)	n.i		1.84 [1.00, 3.36]	0.0487	n.i		1.44 [0.95, 2.18]	0.0825
Serum bicarbonate	1.05 [1.00, 1.11]	0.0702	n.i		0.94 [0.89, 0.99]	0.0172	n.i	
Serum hemoglobin	n.i		n.i		n.i		n.i	
Non-fasting status (ref fasting)	1.19 [0.80, 1.78]	0.3884	1.28 [0.79, 2.07]	0.3223	0.94 [0.65, 1.37]	0.7611	0.90 [0.64, 1.28]	0.5555

Multivariable logistic regression for lipids (binary). Variables remaining significant at the 0.1 level after backward selection included, fasting status forced in the model

*n.i.* not included, *OR* odds ratio, *CI* confidence interval, *ref* reference category, *UACR* urinary albumin/creatinine ratio

## Discussion

In this largest pediatric CKD cohort investigated to date, a characteristic pattern of dyslipidemia involving all lipid fractions, but mainly TG, was highly prevalent irrespective of CKD stage or fasting status and significantly associated with other cardiovascular risk factors.

### Prevalence and type of dyslipidemia

Dyslipidemia was observed in 72% of patients. This prevalence is considerably higher than reported in two other large pediatric cohort studies: 45.2% in the CKiD study (*n* = 391; USA) [4] and 61.5% in the KNOW-Ped CKD study (*n* = 437; South Korea) [5].

However, a smaller cross-sectional study from India (*n* = 80) found a similar prevalence of 73.8% [19]. Many patient characteristics are similar in these cohorts: age (about 11–12 years), a preponderance of boys (60–70% boys), and primary kidney disease CAKUT (about 70%); however, eGFR was lower in our study (26 vs. 43 and 55 ml/min/1.73 m^2^) and the percentage of patients with nephrotic range proteinuria was higher (27% vs. 12% and 15.7%) when compared to the U.S. and Korean studies, respectively. Therefore, a higher degree of proteinuria and a lower eGFR are likely important factors contributing to the high prevalence of dyslipidemia observed in our study. Indeed, proteinuria and eGFR were among the variables significantly associated with dyslipidemia in the multivariate analysis.

As in the other cohort studies, high TG levels were the most common abnormality, but the prevalence was much higher in the present study (54.7%) than in the CKiD study (32%) and the KNOW-Ped CKD study (15.2%).

There were no significant differences between CKD stages in the prevalence of abnormal lipid levels and prevalence and type of dyslipidemia. Isolated dyslipidemia (32.3%), most often in the form of hypertriglyceridemia, was less common than multiple dyslipidemia (39.4%).

### Factors associated with lipid levels and dyslipidemia

The baseline TG levels were independently associated with male sex, eGFR, BMI-SDS, a diagnosis of glomerular disease, a shorter time of CKD, diastolic blood pressure, serum hsCRP and serum albumin levels, and, in contrast to all other measured lipids, the fasting status.

The logistics of implementing *fasting conditions* in pediatric studies can be challenging. In our study, patients were asked to be fasting, and fasting status was documented at all clinic visits. In the Korean KNOW-Ped CKD study, children were apparently not fasting [5], and in the CKiD study, fasting status was unknown in 29% of the children [4]. However, in a study from India reporting a similar prevalence of dyslipidemia, all children were investigated in the fasting status. As expected, we found that triglycerides, but not other lipids, were affected by fasting; however, fasting status had no significant effect on dyslipidemia status in the multivariate analysis. Of note, the non-fasting state may in fact better reflect lipid metabolism under real-world conditions. For practical reasons and for cardiovascular risk prediction [20], several European Medical Societies have recommended that non-fasting blood samples be routinely used for the assessment of plasma lipid profiles in adult patients [21]. While unmeasured variables such as differences in genetic factors or dietary habits between fasting and non-fasting patients may have played a role, our findings suggest that lipid measurements in the fasting state have only limited relevance for identifying dyslipidemia in children with CKD.

Multivariate analysis showed that the *serum albumin level* was the only variable significantly associated with all lipid measurements and thus, in the case of hypoalbuminemia, with an atherogenic constellation of lipids, i.e., higher levels of CHOL, LDL-C, and TG and lower HDL-C. This cannot be explained by malnutrition, inflammation, or proteinuria since UACR, hsCRP, and BMI-SDS were included as covariates. While to our knowledge no studies have been performed in humans to investigate the pathophysiology of this association in non-nephrotic CKD, animal experiments have shown that hypoalbuminemia has a strong inhibiting effect on lipoprotein lipase, independent of proteinuria [22]. It has been shown that lipoprotein lipase activity is reduced in hypoalbuminemia in the absence of proteinuria due to defective endothelial binding [23]. Diminished lipolysis of very low-density lipoprotein (VLDL) with an increase in relatively cholesterol-rich VLDL remnants and diminished formation of HDL cholesterol has been demonstrated as a characteristic disturbance of lipoprotein metabolism in CKD [2]. Hence, an inhibiting effect of lower serum albumin levels on lipolysis of TG-rich lipoproteins would increase the serum levels of TG-rich lipoproteins and explain the associations found in our study, i.e., strong impact on TG and HDL-C and a weak association with CHOL and LDL-C.

Hypoalbuminemia is a marker of inflammation in several disease states and is further aggravated by urinary losses of albumin in nephrotic patients; it has been confirmed as a principal risk factor for the progression of CKD and cardiovascular disease in CKD patients [24, 25].

The degree of *proteinuria* was also significantly associated with lipid levels and the presence of dyslipidemia, respectively. For reliable quantification of proteinuria, measurement of the albumin/creatinine ratio in spot urine is recommended in current guidelines, acknowledging the greater sensitivity, less variability, and better standardization of this method compared to the protein/creatinine ratio [24]. Especially, low concentrations of urine albumin are better detected with the UACR, and large cohort studies have shown that it accurately predicts kidney and cardiovascular risks [14]. Accordingly, we have used the albumin/creatinine ratio in spot urine for proteinuria quantification and used a cut-off for nephrotic range proteinuria in children (1.1 g/g), which should be validated in future studies of children with proteinuria.

*Serum bicarbonate levels* were associated with higher levels of CHOL and LDL-C. This was also noted in previous studies in adults [25, 26]. Higher levels of total and LDL cholesterol could reflect better nutrition with correction of metabolic acidosis [27]. However, a potential increase in serum cholesterol, i.e., atherogenic lipoproteins (presumably VLDL remnants and modified LDL), adds to the uncertainties of bicarbonate supplementation [28]. *Gender differences* in CKD patients have not been much investigated [29]. In our study, the female sex had a significant association with lower TG and higher HDL-C levels. In contrast, high TG levels were more often found in boys in the CKiD study [30].

Time since the diagnosis of CKD (stage 2), a variable reflecting *CKD duration*, is likely a measure of progression or alternatively, late referral to tertiary centers, although the latter is not common in European children; it was associated with lower TG and higher HDL-C levels. The association of *diastolic* (but not systolic) *blood pressure* with TG levels (but not other lipids) remains unclear and should be further investigated in clinical studies.

We found significant associations of *BMI* with CHOL, LDL-C, and TG levels at baseline and with dyslipidemia status for CHOL and TG. The strong association of BMI with elevated TG levels was also found in all previous cohort studies, although BMI distribution was different: the CKiD study and the study from India had a much higher percentage of obese patients (both 28%), whereas the percentage of overweight patients (18%, 20%) was like ours. The study from Korea had only 7% overweight patients and, like our study, 5.1% obese patients while reporting a similar high prevalence of hypertriglyceridemia as in our study. Mean TG levels were clearly elevated across all categories of BMI and highest in overweight and obese children. These data suggest that TG levels are elevated in most CKD patients regardless of BMI category but further increase in overweight and obese individuals.

Although overweight/obesity is associated with inflammation and lack of physical activity, it should be noted that these variables showed independent significant associations with serum lipid levels and dyslipidemia. *Physical activity* was much lower in patients with nephrotic range proteinuria, but the BMI-SDS was similar to other patients, further suggesting an independent effect of physical activity, which was associated with hypercholesterolemia and hypertriglyceridemia in this study. Also, in contrast to the positive association with BMI, *hsCRP* had a negative impact on all baseline lipid levels; it was found to be associated with a lower risk for hypercholesterolemia and a higher risk for low HDL-C levels. This is in line with a large cross-sectional analysis of adults with mild to moderate CKD that found lower levels of CHOL and LDL-C (but not TG or HDL-C) in patients with increased hsCRP (or IL-6) levels [31]. Although not many patients were underweight in our study, these associations could reflect malnutrition, which may not be tightly linked to BMI [32].

### Pathogenesis

Previous studies have revealed a multifactorial pathogenesis of dyslipidemia in patients with CKD [2]. High TG levels, the hallmark of CKD-induced dyslipidemia, are caused by increased hepatic production of VLDL accompanied by diminished catabolism by lipoprotein lipase and hepatic lipase [33]. Additional mechanisms have been demonstrated in nephrotic syndrome, where pronounced hyperlipidemia is driven by massive proteinuria. It could be shown that circulating glycoprotein angiopoietin-like 4 (Angptl4), in response to nephrotic range proteinuria, reduces proteinuria and also inactivates endothelium-bound lipoprotein lipase resulting in impaired lipolysis and hypertriglyceridemia [34]. Thus, Angptl4 provides a molecular link between proteinuria and hypertriglyceridemia and could explain the presence of dyslipidemia in patients with near-normal serum albumin levels (as in the present study). In addition, there is emerging evidence for the role of inflammatory cytokines in the pathogenesis of dyslipidemia in glomerular disease [35]. It could be shown that interleukin-13 (IL-13) induced changes in hepatic cholesterol metabolism in a rat model of minimal change disease leading to hypercholesterolemia preceding the onset of proteinuria [36]. Accumulation of TG-rich lipoproteins in CKD leads to enrichment of all lipoprotein fractions with TG [37] and increased plasma levels of bioactive lipid components, many of which are subject to oxidation, resulting in profound changes in free fatty acid profiles and complex lipids [38, 39]. In fact, modifications in all components of lipoproteins induced by the uremic milieu can promote pro-inflammatory and pro-atherogenic processes and oxidative stress in the kidney (reviewed in [40]). Emerging data from prospective cohort studies confirm the atherogenic potential of dyslipidemia in children with CKD: data from the CKiD study shows that dyslipidemia is associated with increased cIMT in children with CKD at 12 months of follow-up [41], and in the 4C study, pulse wave velocity over time was associated with LDL-C among other risk factors [42].

It is a limitation of our study that more qualitative and detailed analytics such as measuring lipoprotein composition and subclasses (such as VLDL remnants) or exploratory studies (lipolytic activity, free fatty acids, Angplt4, oxidative modifications, etc.) were not performed. Most of our study population was Caucasian, limiting the relevance of our findings for other populations. The potential effect of some antihypertensive drugs (diuretics, beta-blockers) on the lipid profile could not be analyzed due to the small number of patients treated with these medications.

## Conclusions and outlook

We found a high prevalence of dyslipidemia with an established atherogenic potential and could identify significant associations with several other cardiovascular risk factors, which raises the question of treatment options. At present, pharmacological treatment of hyperlipidemia in children with CKD is hardly considered. The current KDIGO guidelines recommend the assessment of lipid profiles; however, lipid-lowering medications are not recommended for this age group, and only lifestyle changes are advised [43]. In contrast, statin treatment is recommended in adult patients [43], where large-scale evidence supports aggressive treatment of dyslipidemia in all stages of CKD (reviewed in [44]). Statin therapy has also been recommended by an expert panel (American Heart Association) for all children with a very high cardiovascular risk > 10 years of age with abnormal LDL-C, in addition to dietary advice and lifestyle modifications [45]. While therapeutic experience in children is limited [46], the anti-inflammatory effect of statins might be reconsidered in treatment decisions in view of the harmful effects of modified lipoproteins as part of the chronic inflammatory process in CKD [47]. The large CANTOS trial has recently shown that in adult CKD patients—in contrast to patients without CKD—already treated with statins, markers of inflammation (hsCRP, IL-6), but not lipid levels, were predictive of major adverse cardiovascular events, suggesting the need for additional anti-inflammatory therapeutic options [48]. At present, further prospective data are needed to support a rationale for choosing and evaluating lipid-lowering medications in children with CKD.

### Electronic supplementary material

Below is the link to the electronic supplementary material.

Graphical abstract (PPTX 86 KB)

Supplementary file2 (DOCX 37.1 KB)

## Data Availability

The data concerning the findings of this study are available from the corresponding author, UQ, upon reasonable request.

## Appendix 4C Study Collaborators

The following principal investigators contributed to the 4C Study:

**Austria**: G. Cortina, Children’s Hospital, Innsbruck; K. Arbeiter, University Children’s Hospital, Vienna. **Czech Republic:** J. Dusek, University Hospital Motol, Prague. **France:** J. Harambat, Hôpital des Enfants, Bordeaux; B. Ranchin, Hôpital Femme Mère Enfant et Université de Lyon; M. Fischbach, A.Zalosczyk, Hôpital de Hautepierre, Strasbourg. **Germany:** U. Querfeld, Charité Children’s Hospital, Berlin; S.Habbig, University Children’s Hospital, Cologne; M. Galiano, University Children’s Hospital, Erlangen; R. Büscher, University Children’s Hospital, Essen; C. Gimpel, Center for Pediatrics and Adolescent Medicine, Freiburg; M. Kemper, UKE University Children’s Hospital, Hamburg; A. Melk, D. Thurn, Hannover Medical School, Hannover; F. Schaefer, A. Doyon, E. Wühl, Center for Pediatrics and Adolescent Medicine, Heidelberg; M. Pohl, Center for Pediatrics and Adolescent Medicine, Jena; S. Wygoda, City Hospital St. Georg, Leipzig; N. Jeck, KfH Kidney Center for Children, Marburg; B. Kranz, University Children’s Hospital, Münster; M. Wigger, Children’s Hospital, Rostock. **Italy:** G. Montini, S. Orsola-Malpighi Hospital, Bologna; F. Lugani, Istituto Giannina Gaslini, Genova; S. Testa, Fondazione Ospedale Maggiore Policlinico, Milano; E. Vidal, Pediatric Nephrology, Dialysis & Transplant Unit, Padova; C. Matteucci, S. Picca, Ospedale Bambino Gesù, Rome. **Lithuania:** A. Jankauskiene, K. Azukaitis, University Children’s Hospital, Vilnius. **Poland:** A. Zurowska, Pediatric and Adolescent Nephrology, Gdansk; D. Drodz, University Children’s Hospital, Krakow; M. Tkaczyk, Polish Mothers Memorial Hospital Research Institute, Lodz; T. Urasinski, Clinic of Pediatrics, Szczecin; M. Litwin, A.Niemirska, Children’s Memorial Health Institute, Warsaw; M. Szczepanska, Zabrze. **Portugal:** A. Texeira, Hospital Sao Joao, Porto; **Serbia:** A. Peco-Antic, University Children’s Hospital, Belgrade. **Switzerland:** B.Bucher, Inselspital, Bern; G. Laube, University Children’s Hospital, Zurich. **Turkey:** A. Anarat, A.K. Bayazit, Cukurova University, Adana; F. Yalcinkaya, University Faculty of Medicine, Ankara; E. Basin, Baskent University Faculty of Medicine, Ankara; N. Cakar, Diskapi Children’s Hospital, Ankara; O. Soylemezoglu, Gazi University Hospital, Ankara; A. Duzova, Y. Bilginer, Hacettepe Medical Faculty, Ankara; H. Erdogan, Dortcelik Children’s Hospital, Bursa; O. Donmez, Uludag University, Bursa; A. Balat, University of Gaziantep; A. Kiyak, Bakirkoy Children’s Hospital, Istanbul; S. Caliskan, N. Canpolat, Istanbul University Cerrahpasa Faculty of Medicine, Istanbul; C. Candan, Goztepe Educational and Research Hospital, Istanbul; M. Civilibal, Haseki Educational and Research Hospital, Istanbul; S. Emre, Istanbul Medical Faculty, Istanbul, H. Alpay, Marmara University Medical Faculty, Istanbul; G. Ozcelik, Sisli Educational and Research Hospital, Istanbul; S. Mir, B. Sözeri, Ege University Medical Faculty; Izmir; O. Yavascan, Tepecik Training and Research Hospital, Izmir; Y. Tabel, Inonu University, Malatya; P. Ertan, Celal Bayar University, Manisa; E. Yilmaz, Children’s Hospital, Sanliurfa. **UK:** R. Shroff, Great Ormond Street Hospital, London.
