# Supplementary material for: Dyslipidemia in children with chronic kidney disease—findings from the Cardiovascular Comorbidity in Children with Chronic Kidney Disease (4C) study
Source: Pediatr Nephrol. 2024 May 8;39(9):2759–72. doi: 10.1007/s00467-024-06389-3 (PMC11272819; doi:10.1007/s00467-024-06389-3)
Supplement: Supplementary file 1 — Graphical abstract (PPTX 86 KB) [file 467_2024_6389_MOESM1_ESM.pptx]

## Slide 1
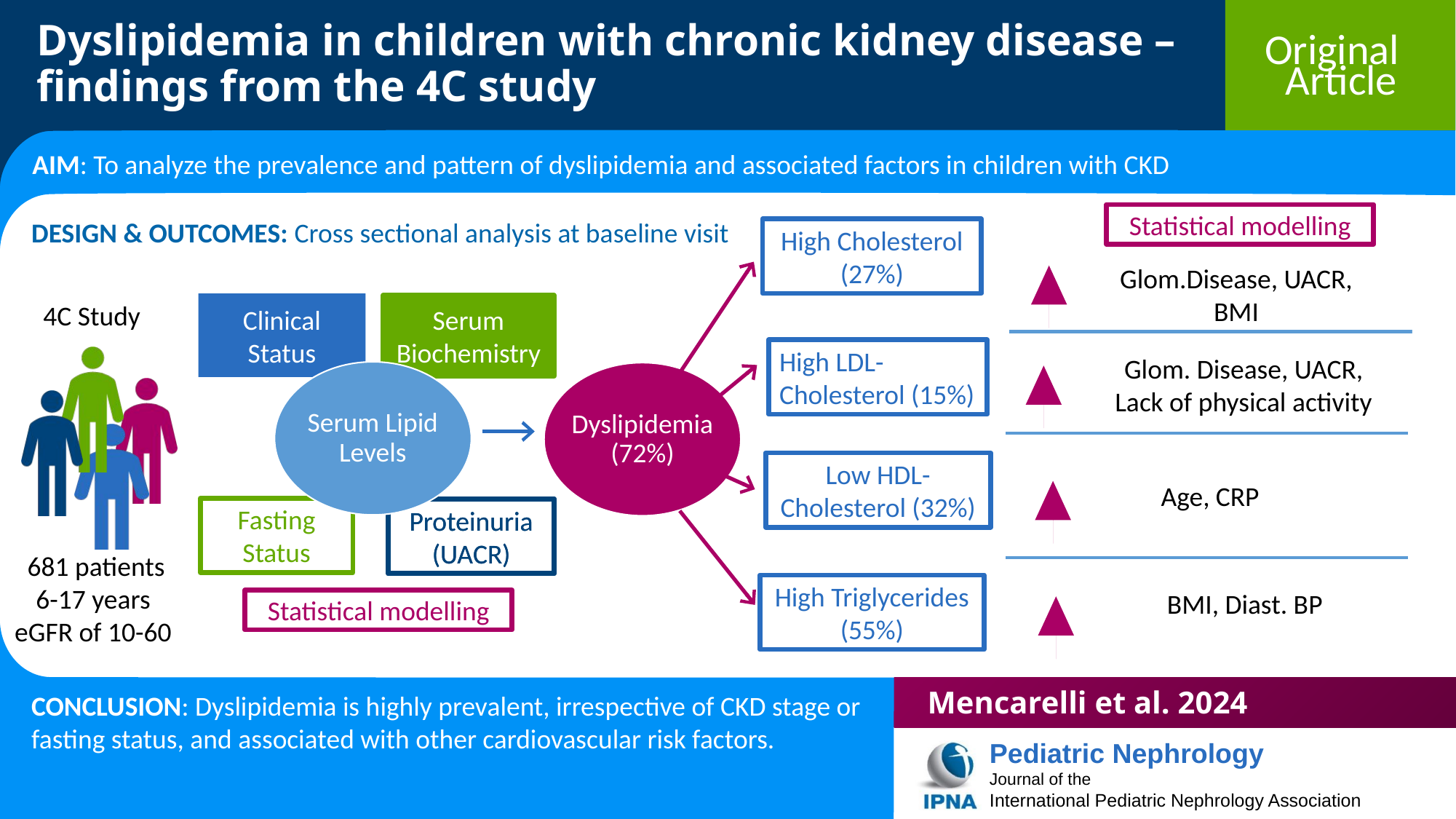

Dyslipidemia in children with chronic kidney disease –
findings from the 4C study
AIM: To analyze the prevalence and pattern of dyslipidemia and associated factors in children with CKD
Statistical modelling
DESIGN & OUTCOMES: Cross sectional analysis at baseline visit
High Cholesterol
(27%)
Glom.Disease, UACR, BMI
4C Study
Clinical Status
Serum Biochemistry
High LDL-Cholesterol (15%)
Glom. Disease, UACR, Lack of physical activity
Serum Lipid Levels
Dyslipidemia (72%)
Dyslipidemia (72%)
Low HDL-Cholesterol (32%)
Age, CRP
Fasting Status
Proteinuria
(UACR)
Proteinuria
(UACR)
681 patients
6-17 years
eGFR of 10-60
High Triglycerides
(55%)
BMI, Diast. BP
Statistical modelling
Mencarelli et al. 2024
CONCLUSION: Dyslipidemia is highly prevalent, irrespective of CKD stage or fasting status, and associated with other cardiovascular risk factors.
