# Supplementary material for: Dyslipidemia in children with chronic kidney disease—findings from the Cardiovascular Comorbidity in Children with Chronic Kidney Disease (4C) study
Source: Pediatr Nephrol. 2024 May 8;39(9):2759–72. doi: 10.1007/s00467-024-06389-3 (PMC11272819; doi:10.1007/s00467-024-06389-3)
Supplement: Supplementary file 2 — Supplementary file2 (DOCX 37.1 KB) [file 467_2024_6389_MOESM2_ESM.docx]

**Dyslipidemia in children with chronic kidney disease - Findings from the Cardiovascular Comorbidity in Children with Chronic Kidney Disease (4C) Study**

**Supplemental Material Table of Contents**

Supplemental Table 1. Reference range for lipid levels in children

Supplemental Table 2. Intercorrelation of lipid values (Pearson correlation coefficients)

Supplemental Table 3. Lipids in the study population and in fasting and non-fasting patients

Supplemental Table 4. Lipids stratified by BMI categories

Supplemental Table 5. Lipids and frequency of abnormal levels at baseline stratified by CKD stage *in fasting* patients (n=265)

Supplemental Table 6. Types of dyslipidemia and frequency at baseline stratified by CKD stage in *fasting* patients (n=265)

**Supplemental Table 1**

Reference range for lipid levels in children *****

| **Category** | **Low** | **Acceptable** | **Borderline-High,** | **High** |
| --- | --- | --- | --- | --- |
| **Total cholesterol** |  |  |  |  |
| 0-19 yrs | — | <170 | 170–199 | ≥200 |
| 20-24 yrs |  | <190 | 190-224 | ≥225 |
| **LDL cholesterol** |  |  |  |  |
| 0-19 yrs | — | <110 | 110–129 | ≥130 |
| 20-24 yrs |  | <120 | 120-159 | ≥160 |
| **Triglycerides** |  |  |  |  |
| 0–9 yrs | — | <75 | 75–99 | ≥100 |
| 10–19 yrs | — | <90 | 90–129 | ≥130 |
| 20-24 yrs |  | <115 | 115-149 | ≥150 |
| **HDL cholesterol** |  |  |  |  |
| 0-24 yrs | <40 | >45 | 40-45 | — |

* All in mg/dl.

Dyslipidemia was defined the presence of one abnormal measured lipid according to the “ Expert panel on integrated guidelines for cardiovascular health and risk reduction in children and adolescents. Summary report”, Pediatrics 2011; 128: S213–S256.

**Supplemental Table 2**

**Intercorrelation of lipid values (Pearson correlation coefficients)**

|  | **Cholesterol** | **HDL-Cholesterol** | **LDL-Cholesterol** | **Triglycerides** |
| --- | --- | --- | --- | --- |
| **Cholesterol**  **p-value**  **n** | 1.00000  679 | 0.24673  <.0001  679 | 0.92755  <.0001  678 | 0.39994  <.0001  678 |
| **HDL-Cholesterol**  **p-value**  **n** | 0.24673  <.0001  679 | 1.00000  681 | 0.15674  <.0001  678 | -0.38069  <.0001  680 |
| **LDL-Cholesterol**  **p-value**  **n** | 0.92755  <.0001  678 | 0.15674  <.0001  678 | 1.00000  678 | 0.18548  <.0001  678 |
| **Triglycerides**  **p-value**  **n** | 0.39994  <.0001  678 | -0.38069  <.0001  680 | 0.18548  <.0001  678 | 1.00000  680 |

**Supplemental Table 3**

Lipids in the study population and in fasting and non-fasting patients

|  | **All** | **Non-fasting** | **Fasting** | **p-value** |
| --- | --- | --- | --- | --- |
| **Total Cholesterol (n)** | 679 | 414 | 265 |  |
| Mean (SD; mg/dl) | 180 (48) | 180 (44) | 180 (54) | 0.953 |
| Median (IQR) | 173 (55) | 175 (56) | 172 (58) |  |
| **HDL - Cholesterol (n)** | 681 | 416 | 265 |  |
| Mean (SD; mg/dl) | 48 (14) | 48 (14) | 47 (15) | 0.627 |
| Median (IQR) | 46 (19) | 46 (18) | 46 (19) |  |
| **LDL - Cholesterol (n)** | 678 | 413 | 265 |  |
| Mean (SD; mg/dl) | 98 (39) | 97 (36) | 100 (44) | 0.354 |
| Median (IQR) | 93 (46) | 92 (45) | 94 (47) |  |
| **Triglycerides (n)** | 680 | 415 | 265 |  |
| Mean (SD; mg/dl) | 147 (86) | 153 (93) | 137 (75) | 0.017 |
| Median (IQR) | 126 (84) | 126 (96) | 123 (80) |  |

**Supplemental Table 4**

Lipids stratified by BMI categories

|  | **All** | **underweight** | **normal** | **overweight** | **obese** | **p-value** |
| --- | --- | --- | --- | --- | --- | --- |
| **Total Cholesterol (n,%)** | 679 | 35 (5.2) | 481 (70.8) | 125 (18.4) | 36 (5.3) |  |
| Mean (SD; mg/dl) | 180 (48) | 177 (47) | 178 (48) | 188 (51) | 187 (41) | 0.126 |
| Median (IQR) | 173 (55) | 171 (64) | 172 (56) | 187 (51) | 186 (43) |  |
| **HDL - Cholesterol (n)** | 681 | 35 | 483 | 125 | 36 |  |
| Mean (SD; mg/dl) | 48 (14) | 48 (12) | 49 (15) | 45 (12) | 43 (17) | 0.019 |
| Median (IQR) | 46 (19) | 46 (15) | 46 (20) | 44 (16) | 40 (22) |  |
| **LDL - Cholesterol (n)** | 678 | 35 | 480 | 125 | 36 |  |
| Mean (SD; mg/dl) | 98 (39) | 94 (38) | 97 (38) | 104 (43) | 98 (35) | 0.286 |
| Median (IQR) | 93 (46) | 92 (40) | 91 (45) | 99 (43) | 95 (38) |  |
| **Triglycerides (n)** | 680 | 35 | 482 | 125 | 36 |  |
| Mean (SD; mg/dl) | 147 (86) | 139 (60) | 139 (79) | 167 (105) | 191 (110) | <.001 |
| Median (IQR) | 126 (84) | 129 (79) | 118 (81) | 138 (99) | 161 (136) |  |

(underweight: BMI-SDS < -2; normal: -2 ≤ BMI-SDS < 1; overweight: 1≤BMI-SDS<2; obese: BMI-SDS ≥2

**Supplemental Table 5**

Lipids and frequency of abnormal levels at baseline stratified by CKD stage *in fasting* patients (n=265)

|  | **All** | **CKD Stage 3** | **CKD Stage 4** | **CKD Stage 5** | **p-value *** |
| --- | --- | --- | --- | --- | --- |
| **Total Cholesterol (n)** | 265 | 84 | 133 | 48 |  |
| Mean (SD; mg/dl) | 181 (54) | 178 (52) | 184 (58) | 172 (44) | 0.352 |
| - normal (n; %) | 192 (72.5%) | 60 (71.4) | 97 (72.9%) | 35 (72.9%) | 0.968 |
| - abnormal (n; %) | 73 (27.5%) | 24 (28.6%) | 36 (27.1%) | 13 (27.1%) |  |
| **HDL - Cholesterol (n)** | 265 | 84 | 133 | 48 |  |
| Mean (SD; mg/dl) | 47 (15) | 50 (14) | 46 (15) | 45 (15) | 0.129 |
| - normal | 173 (65.3%) | 61 (72.6%) | 84 (63.2%) | 28 (58.3%) | 0.194 |
| - abnormal | 92 (34.7%) | 23 (27.4%) | 49 (36.8%) | 20 (41.7%) |  |
| **LDL - Cholesterol (n)** | 265 | 84 | 133 | 48 |  |
| Mean (SD; mg/dl) | 100 (44) | 98 (43) | 104 (48) | 92 (32) | 0.195 |
| - normal | 220 (83.0%) | 69 (82.1%) | 106 (79.7%) | 45 (93.8%) | 0.082 |
| - abnormal | 45 (17.0%) | 15 (17.9%) | 27 (20.3%) | 3 (6.3%) |  |
| **Triglycerides (n)** | 265 | 84 | 133 | 48 |  |
| Mean (SD; mg/dl) | 137 (75) | 123 (65) | 146 (78) | 138 (79) | 0.094 |
| - normal | 123 (46.4%) | 46 (54.8%) | 55 (41.4%) | 22 (45.8%) | 0.155 |
| - abnormal | 142 (53.6%) | 38 (45.2%) | 78 (58.6%) | 26 (54.2%) |  |

CKD stage was modelled as a categorical variable. *p-value is based on the Chi-squared test for

dichotomized lipid variables (normal vs. abnormal) and on ANOVA for continuous lipid variables.

**Supplemental Table 6**

Types of dyslipidemia and frequency at baseline stratified by CKD stage in *fasting* patients (n=265)

|  | **All** | **CKD Stage 3** | **CKD Stage 4** | **CKD Stage 5** | **p-value** |
| --- | --- | --- | --- | --- | --- |
| Dyslipidemia (n) |  |  |  |  |  |
| - no dyslipidemia | 70 (26.4%) | 26 (31.0%) | 29 (21.8%) | 15 (31.3%) | 0.232 |
| - dyslipidemia | 193 (73.6%) | 58 (69.0%) | 104(78.2%) | 33 (68.8%) |  |
| Isolated vs multiple dyslipidemia |  |  |  |  |  |
| - no dyslipidemia | 70 (26.4%) | 26 (31.0%) | 29 (21.8%) | 15 (31.3%) | 0.430 |
| - isolated dyslipidemia | 91 (34.3%) | 29 (34.5%) | 49 (36.8%) | 13 (27.1%) |  |
| abnormal lipid |  |  |  |  |  |
| - Isolated high Chol | 15 (16.5%) | 8 (27.6%) | 6 (12.2%) | 1 (7.7%) | 0.381 |
| - Isolated low HDL | 28 (30.8%) | 8 (27.6%) | 15 (30.6%) | 5 (38.5%) |  |
| - Isolated high Tri | 48 (52.7%) | 13 (44.8%) | 28 (57.1%) | 7 (53.8%) |  |
| Multiple dyslipidemia | 104 (39.2%) | 29 (34.5%) | 55 (41.4%) | 20 (41.7%) |  |
| Number of abnormal lipids: multiple dyslipidemia |  |  |  |  |  |
| - 2 | 60 (57.7%) | 17 (58.6%) | 31 (56.4%) | 12 (60.0%) | 0.626 |
| - 3 | 35 (33.7%) | 11 (37.9%) | 17 (30.9%) | 7 (35.0%) |  |
| - 4 | 9 (8.7%) | 1 (3.4%) | 7 (12.7%) | 1 (5.0%) |  |
